# The influence of sex-specific factors on biological transformations and health outcomes in aging processes

**DOI:** 10.1007/s10522-024-10121-x

**Published:** 2024-07-13

**Authors:** Yongyin Huang, Hongyu Li, Runyu Liang, Jia Chen, Qiang Tang

**Affiliations:** Heilongjiang University of Chinese Medicine, Harbin, 150040 Heilongjiang China

**Keywords:** Sex differences, Aging, Longevity, Sex chromosomes, Sex hormones

## Abstract

The aging process demonstrates notable differences between males and females, which are key factors in disease susceptibility and lifespan. The differences in sex chromosomes are fundamental to the presence of sex bias in organisms. Moreover, sex-specific epigenetic modifications and changes in sex hormone levels impact the development of immunity differently during embryonic development and beyond. Mitochondria, telomeres, homeodynamic space, and intestinal flora are intricately connected to sex differences in aging. These elements can have diverse effects on men and women, resulting in unique biological transformations and health outcomes as they grow older. This review explores how sex interacts with these elements and shapes the aging process.

## Introduction

Sex-specific differences in longevity and senescence are observed in various species (Lemaître et al. 2020; Bronikowski et al. 2011), and the question of such differences remains among the list of knowledge gaps in the study of the biology of aging. The gaps in knowledge on aging can be categorized into three main areas: evolutionary longevity, biological survival and mortality aspects, and aging processes and phenotypic heterogeneity (Rattan 2024). Each of these categories addresses specific issues, with sex-specific differences being closely linked to species-specific regulatory genes, gene copy number variations, differential metabolic regulators, and metabolic specificity. Therefore, in order to understand healthy longevity, it is crucial to focus on both the qualitative and quantitative assessment of the complex and interconnected metabolic, physiological, and psychosocial markers of health that are influenced by sex-specific differences in aging (Rattan 2024). This paper discusses the importance of sex differences in aging and longevity, with reference to animal models and human studies, but it focuses on sex-specific aging in humans.

There are sex differences in the aging process, with women generally having longer lifespans than men. The database on Human Mortality (www.mortality.org) offers comprehensive historical demographic data for 41 nations, with Sweden boasting the most extensive records. In Sweden, women tend to have a slightly longer life expectancy than men, and this difference becomes more pronounced at age 50 compared to birth (Austad and Bartke 2015). Women demonstrate clear survival advantages in early life, later life, and throughout their life cycle, as validated by birth and death records from multiple countries throughout history. Although these differences are widely acknowledged, there is significant debate surrounding the underlying mechanisms. However, it is interesting to note that women often experience poorer health in late life, a phenomenon known as the male–female health-survival paradox (Gordon et al. 2017). One possible explanation for this paradox is that many of the inflammatory diseases that contribute to female mortality are chronic and have lower death rates compared to acute conditions (‘‘GBD 2016 Disease and Injury Incidence and Prevalence Collaborators" 2017). Additionally, the prevalence of frailty, a condition associated with increased vulnerability and decreased resilience, tends to be higher in older women than in men (Della Peruta et al. 2023). This sex difference in frailty has been consistently observed and supported by a meta-analysis (Gordon et al. 2017), providing further evidence of poorer health among older women. It is important to differentiate between longevity and healthy longevity, as the incidence of diseases is not necessarily negatively correlated with longevity. To gain a comprehensive understanding of health and disease, it is crucial to consider the influence of sex as a variable in research and analysis. By taking sex into account, we can illuminate the intricate connections among biological, societal, and ecological elements that lead to the noted sex differences in aging and health results.

Unfortunately, there is a scarcity of research information on sex differences in the aging process, partially attributed to persistent biases favoring male-centric perspectives in research. For example, the field of coronary artery disease diagnosis has predominantly progressed through research focused on men, resulting in a significant funding difference for the study of coronary artery disease in women (Holdcroft 2007). The bias towards male subjects in medical research may be influenced by historical factors, as researchers tend to replicate previous studies that have primarily involved males (Söderström 2001). It is crucial to address these biases and improve efforts toward sex-inclusive research to advance our understanding of sex differences in health, aging, and lifespan across various species.

Presently, the prevailing theories attribute the genetic basis of sex-related aging differences mainly to the differential inheritance of sex chromosomes and mitochondria. However, the true variances in human sex may stem from an amalgamation of sex hormones and various elements including mitochondria, telomere length, gut microbiota, epigenetic modifications, inflammation, and immune reactions. This culminates in two distinct biological systems for males and females, leading to differences in disease susceptibility and thereby influencing the aging trajectory. This review examines recent research on key mechanisms underlying sex differences in aging, focusing on genetic factors like sex chromosomes, telomeres, mitochondria, and epigenetics. It also delves into the roles of sex hormones, homeodynamic space, and gut microbiota in these age-related sex differences.

## Sex Chromosomes

The primary factor contributing to individual sex differences is the presence of sex chromosomes. The inherent differences in sex chromosomes give rise to distinct phenotypes, thereby causing variations in gene expression and epigenetic inheritance (Arnold and Burgoyne 2004). These variances serve as the foundation for initial developmental sex bias in individuals.

### The ‘‘Unguarded X’’ hypothesis

The asymmetric inheritance of sex chromosomes corresponds with the theory of sexually antagonistic pleiotropy. This theory suggests that alleles advantageous to one sex might be detrimental to the other (Tower 2006). Y chromosome genes are exclusively passed down and optimized in males, while X chromosome genes can be optimized in both sexes. It can be said that the phenotypic effects of harmful alleles on the X chromosome may be masked by normal alleles in females, who have a second X chromosome, unlike males who lack this compensatory mechanism. This mechanism is referred to as the "unguarded X" hypothesis (UXh) or the heterogametic sex hypothesis (Tower 2006). A recent study, performing a meta-analysis on lifespan data from 99 families and 229 species, provided conservative support for the UXh mechanism. The study found that individuals of the heterogametic sex generally have significantly shorter lifespans than those of the homogametic sex (Xirocostas et al. 2020). Furthermore, the data revealed that males, when the heterogametic sex, have lifespans 20.9% shorter than females. Conversely, when females are the heterogametic sex, their lifespans are 7.1% shorter than males (Xirocostas et al. 2020). The need for further explanation of the sex difference in lifespan is suggested, with the aforementioned evidence providing indirect support for the UXh mechanism. Sultanova et al. conducted a meta-analysis of sex-specific survival rates and mean age data for 136 animal species (Sultanova et al. 2023). Their analysis showed that the X chromosome size affects the survival difference between sexes. As the length of the X chromosome increases, females tend to have longer lifespans relative to males, aligning with the UXh mechanism’s effects. However, in various bird species, the Z chromosome’s size, compared to autosomes, does not relate to survival rate differences between sexes (Sultanova et al. 2023). This suggests species-specific chromosomal mechanisms in the UXh, especially between mammals and birds.

Theoretically, it is hypothesized that sex-specific inbreeding decline affects males less than females, due to the buildup of harmful recessive mutations on the sex chromosomes. However, some studies contradict this hypothesis, showing that males from inbred lines live longer than those from outbred lines after extreme inbreeding (Bilde et al. 2009). This suggests that the severity of inbreeding decline is related to the degree of inbreeding. Male longevity is negatively correlated with fitness due to inbreeding, where fitness to reproduce is reduced in both sexes. Furthermore, sex differences in inbreeding decline vary, with susceptibility to inbreeding degradation depending on the species, traits, and experimental conditions. A meta-analysis of 321 studies showed minimal differences in inbreeding depression between sexes, with females experiencing slightly higher levels than males (Vega-Trejo et al. 2022). Generalizing this effect appears unconvincing as the majority of experimental subjects were arthropods, with a notable lack of evidence from other species and minimal variance attributed to sex within the dataset (Vega-Trejo et al. 2022). Additionally, Vega-Trejo et al. found no interaction between differences in lifespan across species and sex, and their findings do not endorse the hypothesis that the UXh mechanism results in a sex-specific decline in inbreeding (Vega-Trejo et al. 2022). While it’s true that most X chromosome-linked genetic diseases (like hemophilia) are more common in males than females, some studies challenging this understanding have emerged, particularly those exploring how sex differences affect longevity (Maklakov and Lummaa 2013). This aspect has received inadequate attention in research on aging and the longevity gap between sexes. Inbreeding decline affects longevity, so research on sex differences in longevity or disease should consider inbreeding factors in animal selection. In addition, Connallon and colleagues contended that the strength of the UXh mechanism impact is contingent upon evolutionary factors, including the frequency of harmful mutations in the genetic material, the portions of the genome linked to the X or Z chromosomes, the typical dominance of detrimental mutations, the sex difference in mutation and selection processes, and the average influence of the mutation on an individual’s overall fitness (Connallon et al. 2022). By analyzing data on sex chromosome size, mutation rates, and fitness effects through a population genetics model, it is shown that the UXh mechanism results in a sex difference in lifespan of 3% or less. This mechanism predicts an average mammalian female survival rate only 1–2% higher, significantly lower than the 20% difference reported in empirical surveys of mammalian lifespan (Connallon et al. 2022).

It can be observed that most of the studies on the effect of the UXh mechanism on longevity are indirect experiments. In addition, there is some discrepancy between the experimental results and the predicted results of the mechanism. Future studies need to be more in-depth to bridge this gap. Perhaps the UXh mechanism does not directly affect lifespan, but plays a more important role in the differences in the incidence of sex-related diseases and thus has an impact on lifespan.

## Toxic Y and Y deficiency

Other data suggest that the UXh mechanism is just one among several on the sex chromosomes, including ‘‘toxic Y’’ and ‘‘Y deletions’’. Toxic Y effect, observed in some species, can lead to reduced survival rates during heterogamete formation due to specific repetitive sequences on the Y chromosome (Nguyen and Bachtrog 2021). The occurrence of Y-chromosome toxicity effect may affect the overall repetitive sequence content of both females and males. Recent data indicates that the epigenetics of the Y chromosome changes over the lifespan. In older male Drosophila, the de-repression and jumping of transposable elements in the Y chromosome can lead to the accumulation of deleterious mutations, genomic instability, and accelerated aging (Marais et al. 2018). Due to high levels of transposable factors, sex chromosomes may promote senescence through the toxic Y effect. A study by Brown et al. found that in aged male Drosophila, repetitive sequences on the Y chromosome are disproportionately misexpressed during senescence, which is linked to the loss of heterochromatin in repetitive elements and a broader loss of repressive chromatin from the centromeric region and the Y chromosome in male flies during senescence (Brown et al. 2020). The toxic Y effect is dependent on the size of the nonrecombining region of the Y chromosome, and this effect predicts a higher survival sex gap. The accumulation of harmful mutations and repetitive elements on the Y chromosome results in decreased male survival (Sultanova et al. 2023).

Postzygotic mutations in normal cells produced and accumulated over the lifetime of a human somatic body, known as somatic mosaicism, are genetic changes in which genetically distinct cellular lineages exist in organisms derived from a single fertilized egg and are not normally inherited by the next generation (Forsberg et al. 2017). Sex chromosome mosaicism escalates with age, and the prevalence of age-related chromosome mosaicism abnormalities is higher in males than females; the underlying mechanisms of this sex difference remain unclear. Cellular mosaicism in somatic tissues for all chromosomes intensifies with age, yet the increase is notably more pronounced for sex chromosomes (Marais et al. 2018). Forsberg concluded from multiple datasets that mosaic loss of chromosome Y (LOY) is prevalent in aging men. LOY affects 1.6% of the human genome, making it the most frequent nonphysiological postzygotic mutation in humans (Forsberg et al. 2017). The research illustrates that the LOY phenotype is not passive in the aging process, indicating a correlation between LOY in blood cells and a heightened vulnerability to different types of cancers. As a risk factor exclusive to males for both cancer and Alzheimer’s disease, LOY in blood could help shed light on the reasons behind the typically shorter lifespan of males compared to females (Forsberg et al. 2017).

In summary, sex chromosomes are crucial in explaining widespread patterns of sex differences in lifespan, with particular emphasis on Y-chromosome toxicity among the three key mechanisms involved. Future research should start investigating the specific loci and mechanisms behind sex chromosome toxicity in the lifespan sex gap, employing direct experimental manipulation to assess the relative number and activity of transposable factors on the sex chromosomes.

## Mitochondria

Mitochondria are crucial for cellular functions like producing energy, undergoing oxidation, and regulating apoptosis. Their role in aging has been characterized as a significant aspect of the aging process (López-Otín et al. 2023). Sex dimorphism affects mitochondrial oxidative capacity, calcium management, and resistance to oxidative stress. Additionally, it influences the occurrence of neuromuscular diseases, encephalopathies, cardiovascular diseases, metabolic disorders, and neuropathies linked to mitochondrial dysfunction (Ventura-Clapier et al. 2017). Mitochondrial function plays an important role in autoimmune myocarditis, predominantly affecting males (Di Florio et al. 2020). The prevalence of heart failure with preserved ejection fraction (HFpEF) is twice as high in women compared to men, and women with this condition tend to have smaller and stiffer hearts than men (Mauvais-Jarvis et al. 2020). Mitochondrial gene expression is higher in women than in men and shows a strong correlation with diastolic function, a critical aspect of HF heart failure (Cao et al. 2022). Mitochondrial-origin genetic disorders impact males more than females (Milot et al. 2017), potentially resulting in higher mortality rates in males.

### The ‘‘Mother’s Curse’’ hypothesis

Mitochondria play a multifaceted role in sexual dimorphism, marked by variations in mitochondrial gene expression, DNA copy number, and both mass and number of mitochondria. These differences contribute to sex-specific variations in metabolism, energy production, and other physiological functions. The "mother’s curse" hypothesis aligns with the maternal inheritance theory of mitochondria, suggesting that mutations accumulating in males originate from the mitochondrial genome. This hypothesis posits that when such mutations are neutral, beneficial, or only mildly harmful to females, it permits the accumulation of male-detrimental mutations in the mitochondrial genome (Milot et al. 2017), and over time, these mutations can lead to adaptive defects in the male genome. A low mitochondrial DNA copy number in cells is linked to accelerated aging, reduced cognitive and physical abilities, and higher mortality rates (Mengel-From et al. 2014). Innocenti and his team identified a notable sex-based asymmetry in nuclear gene expression patterns through microarray analysis, examining whole-gene variations in Drosophila melanogaster strains that solely differed by the origin of their mitochondrial genomes. Mitochondrial genes exert a weak influence on the expression of the female-specific transcriptome and a strong influence on male gene expression, accounting for about 10% of all genes tested (Innocenti et al. 2011). In Drosophila and mammals, female mitochondria consume more oxygen for diphosphate supply and possess a higher mitochondrial DNA copy number than males. However, females have a lower survival rate than males in simulated Drosophila environments (Ballard et al. 2007). In fact, this does not imply that maternally inherited mitochondrial DNA is a primary biological mechanism for sex differences in longevity and aging. The complexity of mitochondrial DNA’s role in sexual dimorphism is evident, and the data on the relationship between aging and mitochondrial DNA damage have limited relevance to sex differences.

### Mitochondrial dynamics

It has been suggested that variations in the number and function of mitochondria may contribute to lifespan differences between sexes (Tower 2006). While there are clear similarities in the parameters affecting aging and mitochondrial metabolism between Drosophila and mammals, significant species-specific differences exist (Ballard et al. 2007). Research has shown a difference in skeletal muscle mitochondrial characteristics based on sex; female rats exhibit greater mitochondrial mass and function compared to male rats, partly attributed to sex hormones (Capllonch-Amer et al. 2014). Estrogen and its receptors play a role in regulating the permeability transition of mitochondria. Sex differences in injury caused by ischemia and reperfusion may be linked to how estrogen regulates calcium levels in mitochondria (Fels and Manfredi 2019). Sex hormones may play a key role in explaining the beneficial effects of females on mitochondrial function and aging. Differences in mitochondrial biogenesis, bioenergetics, and morphology between males and females may start to manifest early in life (Khalifa et al. 2017). With age, mitochondrial glutathione decreases up to 50% in several tissues, a decline more pronounced in female mice. This decrease results from an enhanced oxidant milieu and a progressive loss of mitochondrial membrane fluidity (Suh et al. 2004; Marí et al. 2020). In the meta-analysis conducted by Junker, a wide range of variability was observed in the literature across four main domains and six subdomains of mitochondrial biology regarding binary sex differences (Junker et al. 2022). It was determined that there were minor to moderate differences between males and females in certain aspects of mitochondrial biology, with the health of mitochondria being found to vary significantly depending on the specific tissue analyzed (Junker et al. 2022). While human aging is known to involve changes in sex hormone levels and mtDNA quality, there is a relative lack of analyses on this topic. The available analyses mostly consist of cross-sectional studies, and there is a dearth of longitudinal studies that examine sex differences in mitochondria throughout the entire lifespan.

## Telomere Abrasion

Telomeres consist of non-coding TTAGGG DNA repeats. Together with associated proteins, they shield the ends of linear chromosomes, preventing chromosome fusion, coding DNA degradation, and the misidentification of ends as breaks (Blackburn 2000). There have been several studies that support the connection between telomere length and age-related mortality (Cawthon et al. 2003; Codd et al. 2021). Cellular senescence becomes apparent in older individuals, where the efficacy of DNA repair mechanisms diminishes during the aging process of the organism. Moreover, telomeres eventually reach a critical length essential for cell survival, which aligns with the natural lifespan limit of human beings (Steenstrup et al. 2017). At birth, there is sexual dimorphism in telomere length, as cord blood cells of female newborns tend to exhibit longer telomeres compared to their male counterparts (Mayer et al. 2006; Factor-Litvak et al. 2016). This sexual dimorphism in telomere length persists in leukocytes throughout life, providing a potential explanation for the longer lifespan observed in females. However, it is important to note that the causal relationship between telomere length and lifespan remains a topic of controversy. A research investigation into the correlation between genetically determined telomere length and health issues related to aging identified a connection between genetically inclined telomere length and increased cancer susceptibility, while showing a decreased risk of coronary heart disease (Kuo et al. 2019). Furthermore, it suggested that telomere lengthening might offer limited health benefits in later life and could elevate cancer risk (Kuo et al. 2019). This suggests that variations in telomere length result from interactions between genes and the environment.

Hägg et al. proposed two explanations for sexual dimorphism in telomere length. One explanation involves mutations in the telomerase gene *DKC1*, located on the X chromosome, which lead to rapid telomere shortening and reduced survival (Hägg and Jylhävä 2021). The gene *DKC1* codes for dyskerin, an essential protein involved in the folding and stabilization of the initial telomerase RNA molecule, as well as in the formation and function of telomerase (Wong and Collins 2006). Telomerase changes, which evolved in response to cellular immortality and an increased cancer risk, decline with age. Another explanation is related to body size, with the larger sex being disadvantaged in terms of cell maintenance, oxidative stress response, and telomere function, as cell volume is associated with growth (Hägg and Jylhävä 2021). There is a stronger association in men between being overweight or obese and telomere length (Wang et al. 2017; An and Yan 2017). The telomerase enzyme can be activated by sex hormones, and sex hormones may provide an explanation for sex differences in telomere length. Differences in the kinds and levels of hormones released by the placenta might play a role in the noted sex variations in average telomere length during birth (Lansdorp 2022). Exogenous androgens are beneficial for patients with disorders of telomere biology (Townsley et al. 2016). Higher endogenous estrogen exposure is associated with shorter relative telomere lengths (Kresovich et al. 2018). Sex differences in telomere length may also be closely associated with the perception of stress. An analysis using information gathered from the Costa Rica Longevity and Healthy Aging Study employed multiple regression with least squares to assess the link between telomere length and the perception of stress. It discovered that sex significantly moderated the stress-LTL relationship (Méndez-Chacón 2022). Individuals under psychological stress and suffering from psychiatric disorders often exhibit shorter telomeres. These differences in telomere length have been found to vary with both sex and age (Wang et al. 2017; Otsuka et al. 2017). A comparison of leukocyte telomere length between 18 unmedicated patients with major depression and 17 controls showed that accelerated aging of leukocyte telomeres was positively associated with lifetime exposure to major depression (Wolkowitz et al. 2011). Dysregulation of the cortisol stress response leads to DNA damage in the telomere cap. There is a significant difference in the relationship between cortisol arousal response and telomere length among men and women, where an opposite pattern is noted (Thomas et al. 2022).

The sex difference in telomere wear rate is significantly influenced by the *DKC1* gene, cell volume, and sex hormones, particularly in individuals under psychological stress; but the causal relationship between the two cannot be explained for the time being. Future research on telomere wear should encompass all relevant factors, including sex, and employ a cross-sectional analysis to dissect each factor’s impact on aging, thereby elucidating the primary mechanisms.

## Epigenetic alterations

Epigenetics covers both heritable alterations in gene activity and expression, in addition to enduring, long-lasting changes in the transcriptional capacity of cells that might not be passed down (de Lima Camillo and Quinlan 2021). Loss of epigenetic information plays an important role in cellular aging and multiple age-related diseases. The malleability of epigenetic mechanisms allows environmental factors to modify the epigenome in a sex-specific way, particularly when nutritional status and sex regulation impact their function.

During the pre-embryonic developmental stages, the genome undergoes extensive demethylation in order to ensure allotropy in embryonic development. The timing of demethylation differs between maternal and paternal genomes, with the paternal genome demethylating rapidly at earlier stages (Dearden et al. 2018). Differences in DNA methyltransferase (DNMT) expression levels are observed in male and female blastocysts, both early and later in development. Changes in DNMT enzymatic function could explain the notable variations in genome-wide DNA methylation (DNAm) patterns between sexes. The impact of sex hormones on DNMT levels is evident, as progesterone has been shown to decrease DNMT 1, 3a, and 3b (Dearden et al. 2018). While certain methylated CpG sites did show an increase with age, there was no distinct sex-specific trend that emerged. In contrast, Tan et al. demonstrated that increased variability of CpG sites with age was associated with male survival (Tan, Mengel-From, and Christensen 2022). Sex-stratified analysis of twin samples revealed that DNAm variability at more than three thousand CpG sites increased with age in male twins. These changes were primarily associated with genes functioning in cancer-related pathways.

## DNAm involved in reprogramming

In neonatal blood, DNAm levels at autosomal CpG sites are lower in males compared to females, with most differences persisting into late childhood (Shin and Janknecht 2007). In females, DNAm plays a crucial role in X chromosome inactivation (Carrel and Willard 2005). X chromosome inactivation involves the stochastic and selective deactivation of one X chromosome copy in female mammalian cells, leading to a differentiated epigenetic state. This inactivation results from DNAm, which silences gene regions on the X chromosome. During early embryonic development, the epigenetic asymmetry between the two parental genomes is reconfigured to achieve cellular pluripotency, facilitating embryonic cell reprogramming (Cantone and Fisher 2013). Epigenetic reprogramming is deemed crucial for preventing the transmission of inappropriate information to subsequent generations (Cantone and Fisher 2013). During cellular reprogramming, female somatic cells face an additional challenge: reactivating the inactivated X chromosome (Kim et al. 2015). This process involves DNA demethylation, which removes methylation marks from the X chromosome to reactivate its gene regions. When comparing the reprogramming efficiency of induced pluripotent stem cells (iPSCs) derived from male and female somatic cells, it is observed that overall DNA demethylation occurs in the middle and late stages of reprogramming. Whole genome bisulfite sequencing revealed more pronounced demethylation overall during reprogramming in female cells compared to male cells (Milagre et al. 2017). Additionally, the degree of overall DNA demethylation might be influenced by high levels of methylation on the X chromosome. While sex influences cellular reprogramming efficiency, it does not render male somatic cells incapable or significantly less efficient at reprogramming. Male and female iPSCs do not differ in terms of reprogramming efficiency and differentiation potential in vivo (Kim et al. 2015). Sex is just one of several factors influencing cell reprogramming efficiency, with other epigenetic regulatory mechanisms also impacting it.

## DNAm regulates the immune system

DNAm differences regulate immune system aspects, with sex-specific differentially methylated regions in autosomes robustly expressed in immune cells (Mamrut et al. 2015). Sulfite sequencing showed that in female SLE patients, the CD40LG promoter had relatively more demethylation and overexpression of CG pairs compared to unaffected controls (Lu et al. 2007). Changes in gene expression related to hormones in innate immune cells are facilitated by significant remodeling of epigenetics following the activation of hormone receptor signaling cascades (Shepherd et al. 2020). Estrogen signaling effects during puberty can be modified by epigenetic mechanisms. One study showed that changes in monomethylated loci have a strong association with the physiological transition of puberty and changes in reproductive hormone levels (Almstrup et al. 2016). Genome-wide DNAm measurement in peripheral blood mononuclear cells showed that differentially methylated probes were found at or near 312 unique genes. These genes, featuring high-affinity estrogen-responsive elements, are enriched in networks related to immune, inflammatory responses, and reproductive hormone signaling (Thompson et al. 2018). This implies a crucial function for the sex-specific methylome established during adolescence in sex hormone communication. Menopause has been linked to epigenetic alterations, especially in DNAm. A total of 77 CpG sites exhibited noteworthy variations in methylation levels in the blood of postmenopausal women with osteoporosis in comparison to that of healthy women of similar age (Cheishvili et al. 2018). There is a commonly acknowledged understanding that as men age, there is a gradual decrease in testosterone levels. And The link between DNAm and testosterone levels during puberty could help explore how epigenetic mechanisms influence the rate of aging through testosterone levels (Shepherd et al. 2020). In a study examining sex differences in inflammation associated with aging, seven genes linked to IL-6 levels were identified, containing CpG sites whose methylation levels correlate with IL-6. This finding suggests that sex-specific immune system dimorphism in older adults can also be partially explained by DNAm (Nevalainen et al. 2015). This implies a complex interaction among epigenetic, transcriptional, and hormonal regulation, where epigenetic mechanisms are foundational to the effects of hormones.

Besides the interactions between sex hormones and epigenetic mechanisms, sex-related behaviors such as smoking, lifestyle choices, perceived stress and pain, and nutritional habits can also lead to epigenetic changes (Mauvais-Jarvis et al. 2020). These changes regulate gene expression and biological phenotypes, potentially influencing an organism’s phenotype and health. To better understand the role of epigenetic mechanisms in the human body, from embryonic development to aging, integrated and longitudinal approaches are needed. These approaches should investigate how epigenetics interacts with the sex hormone environment and the immune system.

## Inflammation and the immune response

### Involvement of sex hormones in the inflammatory response

Inflammation poses a genuine threat to both the speed of the aging progression and the development of age-related conditions (Franceschi et al. 2018). Inflammation is characterized by an imbalance in systemic cytokine secretion, and this imbalance promotes disease susceptibility and exacerbates chronic conditions, thereby elevating morbidity and mortality risks among the elderly (Olivieri et al. 2023). Additionally, a causal relationship exists between immunity and the elevated prevalence of frailty due to aging. Numerous studies document the relationship between molecules associated with the frailty phenotype and markers of inflammation (Fulop et al. 2015).Elevated plasma levels of IL-6 have been recognized as a predictive biomarker of all-cause mortality in the elderly population (López-Otín et al. 2023). Studies have shown sex differences in the genomic regulation of inflammatory responses. Specifically, expression levels of 62 genes were found to correlate with IL-6 levels in older men, which correlates with DNAm, but no such association was found between PBMC gene expression profiles and plasma IL-6 levels in women (Nevalainen et al. 2015). Immune aging occurs earlier in men than in women, but this is not necessarily a negative outcome. Aging might prompt the immune system to regulate and adapt, enhancing its responsiveness to environmental challenges, and not just to an eventually terminal deterioration of the immune system (Fulop et al. 2017). However, the immune system efficiency advantage females possess during their reproductive period diminishes after this period ends. IL-1β, IL-6, and TNF-α are significantly higher in males compared to females, a tendency that can be partially explained by sex hormones (Bernardi et al. 2020). Estrogen has anti-inflammatory effects on endothelial and immune cells, promoting cardioprotection in premenopausal women. 17β-estradiol exhibits anti-inflammatory activity, guiding the inflammatory process towards an IL-10-dependent "acquired inactivation" phenotype, crucial for tissue remodeling and restoring homeostasis (Acosta-Martínez 2020). The anti-inflammatory effects of estradiol depend on its concentration. Low physiological levels enhance the pro-inflammatory abilities of human and mouse macrophages and monocytes. In contrast, high levels, typical of late pregnancy, boost their anti-inflammatory function (Shepherd et al. 2020). Sex bias in HFpEF is also strongly associated with sex hormones and inflammation; premenopausal women exhibit less cardiac inflammation than men, resulting in reduced fibrosis (Gordon et al. 2017). Inflammation-related sex differences may serve as a junction among various factors, aiding in the identification and refinement of sex-specific biomarkers for aging trajectories (Olivieri et al. 2023). This could facilitate tailored intervention protocols for sex differences in aging-related diseases.

### Innate and Adaptive immune responses

The immune system is significantly impacted by sex, as evident from the broad distribution of sex hormone receptors in different cell types and the diverse expression of genes located on the X chromosome (Dunn et al. 2024). Epidemiological data that is accessible suggests that variations in sex are significant factors in immune responses to viral, autoimmune, and tumor antigens ("GBD 2016 Disease and Injury Incidence and Prevalence Collaborators" 2017). Females generally display stronger innate and adaptive immune responses. In terms of innate immunity, females show higher phagocytic activity in neutrophils, macrophages, and other antigen-presenting cells compared to males (Olivieri et al. 2023). Infected males demonstrate a higher M1/M2 macrophage ratio than females (Deny et al. 2022). After the age of 65, genomic differences between the sexes widen, with males exhibiting higher innate and proinflammatory activities and lower adaptive immune responses (Márquez et al. 2020). These sex differences in immune responses are largely due to sex chromosome gene expression and sex hormones. The variation in X-linked gene expression is a key factor in the immune response differences between sexes, and numerous genes on the X chromosome code for proteins associated with immune responses, including toll-like receptors (TLRs), interleukin 2 receptor, and transcription factors like FOXP3, which regulate immune response ("GBD 2016 Disease and Injury Incidence and Prevalence Collaborators" 2017). Compared to men, women are less susceptible to infections by bacterial, viral, and parasitic pathogens (Shepherd et al. 2020). Most immune cells have estrogen and progesterone receptors. 17-β estradiol boosts cell-mediated and humoral immune reactions, while progesterone demonstrates anti-inflammatory impacts. In contrast, androgens tend to suppress the immune system (Wilkinson et al. 2022). This immunosuppressive response gives testosterone a protective role in adult influenza pathogenesis (Vom Steeg et al. 2016), which is mediated by an immune response that is initiated and maintained by controlling infection. Female adult rodents, on the other hand, show slower repair of lung damage and higher lung inflammation compared to males (Di Florio et al. 2020).

### Autoimmunity

Compared to men, women have a higher immunoreactivity to both auto and non-auto molecular patterns (Olivieri et al. 2023). Multiple autoimmune sensitivities correlate with conditions of fluctuating androgen and estrogen levels. Older men with relatively low serum androgen levels have a higher incidence of rheumatoid arthritis (Gubbels Bupp and Jorgensen 2018). Analysis of transcriptomic data indicates that the majority of distinctions in adaptive immune responses based on sex are primarily influenced by variations in B-cell genetic transcription (Olivieri et al. 2023). Estrogen plays a crucial role in B cells by modifying their development and decreasing lymphangiogenesis (Hill et al. 2011); estradiol increases immunoglobulin synthesis in mature B cells (Khan and Ansar Ahmed 2015). Age-associated B cells (ABCs) progressively rise with increasing age and are associated with autoimmune conditions such as systemic sclerosis and rheumatoid arthritis (Mouat et al. 2022). Upon activation, ABCs secrete large amounts of IL-10, driving the initial CD4+ T cell population towards Th17 differentiation (Olivieri et al. 2023). Increased TLR7 expression might contribute to the higher susceptibility of females to B cell-mediated autoimmune diseases, such as SLE (Wilkinson et al. 2022). In contrast, males’ relative protection against CNS and thyroid autoimmunity may be due to the varying effects of sex hormones on the expression of the autoimmune regulator (AIRE) (Wilkinson et al. 2022). Multiple androgen-responsive elements have been identified in the AIRE promoter, with the androgen promoter specifically localized to the AIRE motif in a manner dependent on androgens (Zhu et al. 2016). Estrogen reduces AIRE expression by modulating CpG methylation at the AIRE promoter (Dragin et al. 2016).

The above discussion highlights how differences in immune response based on sex influence disease susceptibility and severity, partly due to sex steroid hormones interacting with the immune system. In addition, we have recognized the role of X-linked genes in determining female bias in autoimmune diseases. However, we have not discussed how the aging of the immune system and increased inflammatory aging are significantly influenced by mitochondrial dysfunction and changes in gut flora (Fulop et al. 2017). These aspects also interact with gender factors to regulate aging, as mentioned in other sections. We need to consider whether immune senescence and inflammatory aging have only negative effects. This remains controversial, and the views of Tamas Fulop et al. may offer a new perspective (Fulop et al. 2017).

## Homeodynamic Space

Aging is marked by a decrease in the ability to adapt, attributed to the gradual breakdown of internal processes. The concept of homeodynamic space plays a crucial role in understanding this phenomenon, as it combines adaptive homeostasis, homeodynamics and allostasis. Homeodynamic space encompasses stress, response, damage control, and continuous remodeling, serving as indicators of an organism’s ability to survive, its longevity, and overall health (Rattan 2024). The quintessential components of the homeodynamic machinery include repair pathways for nuclear and mitochondrial DNA, the processes for sensing and responding to intra- and extra-cellular stressors, the pathways for protein repair and the antioxidative and enzymic defences against reactive oxygen species, and more (Rattan 2007). Homeodynamics is a longevity assurance mechanism, and survival beyond the species’ basic lifespan inevitably results in a gradual accumulation of random molecular damage (Rattan 2007).

Certain modifications to the homeodynamic range are facilitated by specific signaling pathways that have the ability to temporarily modify transcription and translation. For instance, mitochondrial dysfunction can initiate "retrograde signaling" events, driving adaptive alterations in nuclear gene expression and metabolic processes via distinct transcription regulators (Shadel and Horvath 2015). Reduced oxidative phosphorylation (OXPHOS) capacity or changes in electron transport chain function can lead to energy deprivation, changes in mitochondrial ROS (mtROS) production, or loss of mitochondrial membrane potential. These changes, in turn, trigger specific stress signaling responses. There is a wealth of evidence supporting the key involvement of mtROS signaling in the overall health of organisms and their ability to adapt to various challenges (Shadel and Horvath 2015). Decreased protein levels in mitochondria, incorrect formation of complex enzymes like OXPHOS and ribosomes, and changes in chaperone functions may result in proteotoxic stress and activate unfolded protein responses in mitochondria (Haynes et al. 2013). Typical pathways for stress response, such as DNA repair and apoptosis, are employed to detect mtROS for activating adaptive homeostatic reactions, rather than the originally identified emergency and cell death responses (Shadel and Horvath 2015).

It is crucial to understand the pathways of sex-based differences in disease presentation and death, which also play a key role in the daily adaptive responses necessary for maintaining cellular balance. Hydrogen peroxide (H_2_O_2_) is currently one of the main components of the study of oxidative stress in endogenous metabolic processes. In vitro cellular studies have found that high concentrations of H_2_O_2_ damage cells, but lower, non-toxic levels can enhance protection by promoting cell proliferation and activating stress-response pathways (Wiese et al. 1995). The amount of H_2_O_2_ signaling induces transcription factors that are required for key adaptive cellular responses involving the insulin-like recepto, AKT pathway and Nrf2 activation (Pomatto et al. 2018). Oxidative stress-related proteases were found to exhibit sex-specific adaptive responses to H_2_O_2_ in Drosophila melanogaster; females adapted better to hydrogen peroxide stress, while males adapted better to paraquat (superoxide) stress (Pomatto et al. 2017). Additionally, both male and female Drosophila lose their adaptation to oxidative stress as they age. For instance, the induction of Lon protease by H_2_O_2_ exposure diminishes with age (Pomatto et al. 2017).

Heat Shock Proteins (HSPs) are ubiquitous evolutionarily conserved but diverse chaperone proteins that serve as the first line of defense against proteotoxic stress. Cellular HSPs exhibit functional diversity in stress defense (Ungelenk et al. 2016). The predominant knowledge of the adaptive cellular response to heat shock in vertebrate cells derives from research conducted primarily on female subjects. Exposure to gentle heat during early stages of development has been found to enhance tolerance to future heat stress in Drosophila (heat stress acclimatization) and slightly prolong lifespan, with males experiencing more significant increases than females (Khazaeli et al. 1997). While overexpressing stress response proteins like HSPs and Superoxide dismutase can lengthen lifespan in invertebrates, it may not fully restore the dynamic, coordinated processes of adaptive responses. In fact, in some cases, overexpressing stress response enzymes has been deleterious. Pomatto et al. suggest focusing on minimizing damage accumulation to prevent the loss of adaptive homeostasis with age, rather than merely inducing chronic overexpression of stress response defenses (Pomatto et al. 2018).

The progressive failure of homeodynamic leads to physiological malfunctions, manifesting in overall functional decline, disease, and ultimately death (Rattan 2007). Thus, rational strategies to slow down aging or to prevent age-related frailty and disease require stimulating and strengthening the individual’s homeodynamic processes.

## Intestinal Flora

Microbial communities are widely distributed across the oral cavity, upper respiratory tract, skin, vagina, and intestines. They constitute a complex and dynamic ecosystem interacting with various body organs, the most studied of which is the intestinal flora. The body’s largest immune organ, the gut, is constantly exposed to environmental influences but manages to maintain tolerance. Disruption of the gut flora can disrupt the immune system equilibrium, resulting in increased activation of the innate immune response and continued inflammation (Markle et al. 2013). This chronic low-grade inflammatory state damages cells and tissues, ultimately promoting many age-related degenerative pathologies and unhealthy aging. The bacteria-to-human cell ratios vary by sex, with males showing a 1.3:1 ratio and females a 2.2:1 ratio (Yoon and Kim 2021). This suggests potential sex differences in the gut microbiome. Gut microbe composition and function change after weight loss, varying by sex and diet; men and women experience different dietary benefits (Cuevas-Sierra et al. 2021), indicating potential sex-specific effects on microbiome regulation.

Gut microbes can metabolize estrogens produced within the body or ingested through food. These resulting metabolites have an impact on the host’s sex hormones, thereby directly controlling bacterial metabolism via steroid receptors like estrogen receptor beta. Members of the gut microbiome interact with steroids and may influence steroid homeostasis in the gut and metabolic activity in the colonic ecosystem. For example, Slackia sp., a common gut microbiota member, can facilitate the interconversion of b-estradiol and estrogen (Gomez et al. 2015). Estrogens not originating in the ovaries are impacted by the gut microbiota and enzymes like β-glucuronidase. Moreover, women experience a significant decrease in fecal β-glucuronidase activity with aging (Walsh et al. 2020). Clostridium scindens, a commensal in the intestine, contains hydroxysteroid dehydrogenase and additional enzymes that play a role in converting glucocorticoids into androgens (Ridlon et al. 2013). Studies have demonstrated a significant correlation between male testosterone levels and the abundance of Acinetobacter, Dorea, Ruminococcus, and Megamonas in the data. Women with higher testosterone levels exhibited an increased presence of Bacillus-like genera and a decreased presence of solidifying genera compared to those with lower levels (Shin et al. 2019). These data suggest that specific gut flora and sex hormones interact, regulating disease outcomes in individuals genetically prone to autoimmune diseases, especially those exhibiting sex differences. Feedback loops between sex hormones and gut microbes drive microbiota expansion, which likely influences autoimmunity by triggering inflammatory or tolerance effects, subsequently impacting the aging process.

Alterations in gut microbiota are age-dependent as well, displaying notable variances in gut microbial structure and richness between individuals reaching one hundred years old and those in early adulthood (Biagi et al. 2010). Sex distinctions within these mechanisms manifest around the same life stages, indicating a correlation between sex and gut flora. However, studies show inconsistent results regarding sex differences in microbial taxa. It has been claimed that sex differences in gut flora become more pronounced in the presence of intestinal infections, with the abundance of anaplasmosis increasing while the abundance of E. coli decreasing with age (Singh and Manning 2016). These differences are most notable in patients with intestinal infections. Lai et al. used ITS analysis to characterize the fungal community structure in the human gut, finding strong associations with age and disease for fungal enterotypes, but no significant links to BMI or sex (Lai et al. 2023). In contrast, Gomez et al. concluded that current data on sex and specific gut microbiota (GM) are unreliable. They suggest that differences in GM composition might stem from living environments and dietary habits, rather than from sex hormones (Gomez et al. 2015).

## Sex Hormone

Masculine development of male fetuses involves the entry of testosterone into the brain and its conversion to estradiol by aromatase. Estradiol is believed to play a crucial role in the early development of the male brain, regulating processes like neurogenesis, cell migration, apoptosis, and the development of key brain regions including the hypothalamus. Sex differences exist in the quantity and functionality of preopiomelanocortin neurons in the hypothalamus’s arcuate nucleus, partially due to the effects of sex steroids on these neurons (Dearden et al. 2018). The male testosterone surge occurs during the first trimester of pregnancy and it alters gene expression and organ organization via epigenetic mechanisms. This plays a critical role in physiological sex differences and disease susceptibility in adulthood (Mauvais-Jarvis et al. 2020). During fetal life, female estrogens are initially produced by the corpus luteum, then by the placenta, and in normal adulthood by the ovaries, adrenal glands, and adipose tissue. The estrogen family, with its two receptors ERα and ERβ, is well known for its protective effects against obesity, type 2 diabetes, and cardiovascular disease. With the onset of menopause and loss of estrogen, the incidence of ischemic stroke begins to increase in women. The prevalence of stroke is greater among females aged 80 and abovethan it is among males within the identical age bracket (Mauvais-Jarvis et al. 2020), highlighting the protective role of endogenous premenopausal estrogens, a finding supported by rodent ischemic stroke models (McCullough et al. 2001).

Sex hormones following puberty have an added impact on cells through epigenetic changes, contributing to differences in cancer based on sex (Mauvais-Jarvis et al. 2020). The involvement of estrogen in the maintenance of energy homeostasis in different tissues may account for the observed sex differences in early life programming susceptibility. Testosterone reduces mitochondrial proliferation, whereas estradiol enhances mitochondrial function and reduces oxidative stress (Capllonch-Amer et al. 2014). Estrogen serves as a defensive force against various illnesses, whereas testosterone may elevate disease susceptibility and reduce lifespan (Clocchiatti et al. 2016). Sex differences in myocarditis are associated with sex hormones, which act not only on immune cells also expressed on cardiac tissue. In women, 17β-estradiol offers some protection to cardiomyocytes, preventing myocardial hypertrophy and fibrosis (Di Florio et al. 2020). Veterinary databases indicate minimal sex differences in lifespan post-sterilization, suggesting sterilization extends lifespan, partly due to sex hormones (Hoffman et al. 2018). Nisi Jiang et al.’s study showed pre-pubertal castration lowers early to mid-term male mortality and lengthens their lifespan to match females’, thus removing lifespan differences between sexes (Jiang et al. 2023). This suggests deeper mechanisms behind testosterone’s effects on longevity warrant further investigation. Dehydroepiandrosterone (DHEA), along with its sulfate form, are the most abundant steroid hormones found in humans. They are converted into testosterone and estradiol in peripheral tissues (Hägg and Jylhävä 2021). Up to 50% of testosterone in older men is derived from DHEA, while in postmenopausal women, DHEA constitutes nearly all estrogen sources (Hägg and Jylhävä 2021). However, DHEA may have more pronounced effects on cognitive function in men (Chen et al. 2018). In the elderly, DHEA has immunomodulatory effects, increasing monocytes, T cells with expressing T cell receptor γ / δ, and NK cells (Rutkowski et al. 2014).

This section only partially discusses the role of sex hormones in age-related diseases; their role has been further elaborated in other sections. Sex hormones play an anti-inflammatory role in immunity, interact with immune systems, regulate DNMT activity impacting epigenetic mechanisms, activate telomerase genes affecting telomere length, are involved in mitochondrial permeability regulation, and interact with intestinal flora to maintain a balanced gut environment and hormone levels. Thus, sex hormones are involved at the genetic, cellular, and systemic levels throughout the body, affecting all aspects of aging-related mechanisms, and there is still a need to focus on the sex-specific aging effects of sex hormones in the future.

## Conclusions

Significant sex differences in aging have been observed in both human and animal studies. The influence of sex on various disease systems indicates that aging is not driven by a single factor; instead, multiple mechanisms interact without a clear pattern. Sex chromosomes determine genetic sex, while epigenetic mechanisms, through DNAm, regulate the inactivation and reactivation of the X chromosome, crucial for sex-specific gene expression. Evidence increasingly shows that sex hormones affect lifespan and interact with the immune system, mitochondrial function, telomere length, and intestinal flora, regulating sex differences from embryonic development to aging and impacting aging-related diseases. Studies on aging, cancer, and development show that molecular pathways, such as PI3K, and tumor suppressors like p53 and retinoblastoma proteins, display sexual dimorphism and require targeting specific to each sex (Mauvais-Jarvis et al. 2020). Therefore, considering sex differences within social, historical, and developmental contexts enhances our understanding of diversity and individual variations in aging. Incorporating sex dimensions in clinical research and practice aids in understanding the varied clinical manifestations and outcomes of diseases between sexes.
